# Aqueous and gaseous plasma applications for the treatment of mung bean seeds

**DOI:** 10.1038/s41598-021-97823-1

**Published:** 2021-10-04

**Authors:** Martina Darmanin, Antje Fröhling, Sara Bußler, Julia Durek, Susanne Neugart, Monika Schreiner, Renald Blundell, Ruben Gatt, Oliver Schlüter, Vasilis P. Valdramidis

**Affiliations:** 1 Department of Food Sciences and Nutrition, Faculty of Health Sciences, University of Malta, Msida, MSD2080 Malta; 2 Quality and Safety of Food and Feed, Leibniz Institute for Agricultural Engineering and Bioeconomy (ATB), 14469 Potsdam, Germany; 3 Division Quality and Sensory of Plant Products, Georg-August Universität Göttingen, 37075 Göttingen, Germany; 4 Leibniz Institute of Vegetable and Ornamental Crops e.V., Grossbeeren, Germany; 5 Department of Physiology and Biochemistry, Faculty of Medicine and Surgery, University of Malta, Msida, MSD2080 Malta; 6 Metamaterials Unit, Faculty of Science, University of Malta, Msida, MSD2080 Malta; 7 Centre for Molecular Medicine and Biobanking, University of Malta, Msida, MSD2080 Malta

**Keywords:** Microbiology, Plant sciences, Materials science, Optics and photonics

## Abstract

Sprouts are particularly prone to microbial contamination due to their high nutrient content and the warm temperatures and humid conditions needed for their production. Therefore, disinfection is a crucial step in food processing as a means of preventing the transmission of bacterial, parasitic and viral pathogens. In this study, a dielectric coplanar surface barrier discharge (DCSBD) system was used for the application of cold atmospheric plasma (CAP), plasma activated water (PAW) and their combination on mung bean seeds. Germination assessments were performed in a test tube set-up filled with glass beads and the produced irrigation water. Overall, it was found that the combined seed treatment with direct air CAP (350 W) and air PAW had no negative impact on mung bean seed germination and growth, nor the concentration of secondary metabolites within the sprouts. These treatments also reduced the total microbial population in sprouts by 2.5 log CFU/g. This research reports for first time that aside from the stimulatory effect of plasma discharge on seed surface disinfection, sustained plasma treatment through irrigation of treated seeds with PAW can significantly enhance seedling growth. The positive outcome and further applications of different forms, of plasma i.e., gaseous and aqueous, in the agro-food industry is further supported by this research.

## Introduction

Cold plasma technology is leading major breakthroughs in addressing a plethora of issues in the agriculture and food sectors, from mitigating produce losses due to pathogens and pests, to enhancing the yields and safety of food^1–4^. Being an ionised gas, cold atmospheric plasma (CAP) constitutes of a complex mixture of active agents, such as UV photons, charged particles, radicals and other reactive nitrogen, oxygen and hydrogen species (RNS, ROS and RHS)^5,6^. These reactive species, individually and/or synergistically, demonstrate microbial inactivation properties while also play a role in promoting germination, rooting and growth of plants^7,8^. Cold plasma processes are most efficient and have the least negative impact on food at atmospheric pressure and low temperatures (generally < 70 °C)^9^.

A plasma system, including its electrode configurations and its process parameters concerning energy supply, working gas and treatment time all affect the composition of the generated plasma and consequently also the antimicrobial and germination efficiency of the plasma treatment^10^. The versatility of plasma set-ups enable unique designs for the precise generation of plasmas and controlled production of acting agents compatible with the needs of the agro-food industry^9^. CAP treatment, having a low impact on the internal product matrix and being overall a resource-efficient application free of water (in case of CAP only), solvents and residues, is considered a potential alternative to conventional chemical disinfection treatments and physical disinfection methods, such as thermal and high-pressure treatment, pulsed electric fields, and ionizing irradiation^11,12^.

The growing popularity of sprout consumption may be attributed to research from nutritional experts revealing the chemistry of sprouting seeds and their biological value in human and animal nutrition^13–15^. Apart from being a significant source of amino acids, proteins, fibre, enzymes, vitamins and minerals fundamental to human health^13^, sprouts also naturally contain a number of bioactive compounds, known as phytochemicals^16^. The principal phytochemicals found in sprouts of the legume family, including alfalfa, mung bean, clover, peas, chickpeas and soybeans are phenolic compounds^16^. A large array of phytochemicals exhibit antioxidant properties, and it has been reported that the presence of phytochemicals in the diet has been shown to provide beneficial effects to human health^17^. Epidemiological data identified an inverse relationship between the intake of phenolic rich food and the rate of chronic diseases such as diabetes, cardiovascular diseases, Alzheimer’s disease, Parkinson’s disease and inflammation^18^. Flavonoids are the most common subtype of phenolic compounds and have the potential to alter lipid metabolism, reduce atherosclerotic lesion formation, improve endothelial function, prevent platelet aggregation and reduce blood pressure^19^. Flavonoid-containing sprouts of cash crops with specific health benefits include buckwheat sprouts, mung bean sprouts, barley sprouts and fenugreek sprouts^20–23^. Flavonoids have a number of protective roles, primarily on vulnerable neurons with a simultaneous ability of stimulating neuronal regeneration in order to maintain and enhance brain function^19^. Furthermore, flavonoids have the ability to quench free radicals to prevent oxidative rancidity, a major cause of food quality deterioration^24^. Chemoprevention is another notable benefit of flavonoids^19,25^.

Disinfection is a crucial step in food processing as a means of preventing the transmission of bacterial, parasitic and viral pathogens and outbreak of food-borne illness from contaminated fresh produce^26^. Sprouts are particularly prone to contamination due to their high nutrient content and the warm temperatures and humid conditions needed for their production^27^. As sprouts are also often consumed raw, disinfection of seeds is obligatory according to the European sprouted seeds association ((2017/C 220/03) in order to ensure that this healthy, inexpensive, convenient food is, above all, consumer safe.

Few studies have assessed, in parallel, cold atmospheric plasma’s properties on microbial inactivation, seed germination and composition of secondary metabolites. Among the different plasma sources cited in the literature, the dielectric barrier discharge (DBD) gains attention as it allows for homogeneous CAP treatment of large volumes that is ideal for seed treatment^28^. Furthermore, the use of air as a process gas is a common trend for cold plasma application on sprouts, notably due to the presence of O_2_ and N_2_, the parent molecules of reactive oxygen and nitrogen species (RONS) that function as the prime antimicrobial agents^29^, with an additional impact on enhancing seed germination^30^. These may include nitrogen oxides (NO^·^ and NO_x_), peroxynitrite (ONOO^−^), atomic oxygen (O), ozone (O_3_), singlet oxygen (^1^O_2_), superoxide anion (O_2_^−^), hydrogen radicals (H^·^), hydroxyl radicals (OH^·^) and/or hydrogen peroxide (H_2_O_2_)^5^. Apart from the direct treatment of seeds with CAP, a significant branch of cold plasma research is being developed in the realm of plasma activated water (PAW). The latter is the product of CAP reacting with water, whereby the ionised gas reacts within or with the surface of water to create reactive species in the water related to the properties of CAP^6,31^. The produced reactive species in PAW could be similarly include atomic oxygen, singlet oxygen, superoxide, ozone, hydroxyl radicals and excited and atomic nitrogen, known as primary species as well as hydrogen peroxide, peroxynitrite, nitric oxide, nitrates and nitrite ions, known as secondary species^32,33^. Zhou et al.^34^ proved that PAW or plasma treated water (PTW) generated with air improved seed germination and seedling growth while it had pronounced antimicrobial properties.

Further efforts at standardising cold plasma technology on food are required in order to determine if cold plasma technology complies with the European Commission’s ‘Novel Food’ regulation (EU) 2015/2283. This should involve research evaluating the safety and quality parameters (i.e., nutrient content, colour, texture and chemical composition) of cold plasma treated food^35^. In this study, a coplanar surface barrier discharge system was used for the application of CAP and PAW treatment on mung bean seeds. The efficacy of the combined application of CAP and PAW on the germination and growth of mung bean seeds was assessed and the antimicrobial functionality of the PAW was also evaluated in order to report its disinfectant capacity. Additional analysis was carried out on the decontamination efficiency of combined CAP and PAW treatment on the natural microbiota within mung bean sprouts. Ancillary analyses were carried out in order to characterise the reactive species in the plasma activated water and to assess the composition of secondary metabolites in the harvested sprouts.

## Results

### Seed germination and growth

#### Individual application of CAP and PAW on mung bean seeds

Preceding the combined CAP and PAW treatment of mung bean seeds, experimental assessment was performed on the CAP and PAW treatments individually. The percentage of mung bean seeds germinated within 96 h, was 97.5% for the control, CAP (air), CAP (N_2_), PAW (air), PAW (N_2_) and PAW (CO_2_) treatments and 90% for CAP (CO_2_) treatment. The growth rate of the sprout stems, expressed as rate constant (*k*) values (1/h) and doubling time (*Dt*), i.e., the time (h) taken for the sprouts to double in length were obtained using the exponential growth equation and reported in Table 1.

**Table 1:** Growth rate (*k*) and doubling time (*Dt*) of mung bean sprouts grown from CAP treated seeds and PAW irrigated seeds.

	Control			CAP (air)			CAP (N_2_)			CAP (CO_2_)		
		CI_upper_	CI_lower_		CI_upper_	CI_lower_		CI_upper_	CI_lower_		CI_upper_	CI_lower_
*k*_1–3_ (1/h)	0.037	0.041	0.034	0.037	0.042	0.032	0.034	0.038	0.030	0.031	0.034	0.030
*Dt*_1–3_ (h)	18.65	20.50	17.03	18.37	21.44	16.48	20.67	23.52	18.27	22.69	24.85	20.81
R^2^	0.9909			0.9817			0.9822			0.9922		
S_yx_	0.2381			0.3811			0.3577			0.1684		

	Control			PAW (air)			PAW (N_2_)			PAW (CO_2_)		
		CI_upper_	CI_lower_		CI_upper_	CI_lower_		CI_upper_	CI_lower_		CI_upper_	CI_lower_
*k*_1–3_ (1/h)	0.038	0.041	0.036	0.034	0.038	0.031	0.036	0.039	0.033	0.036	0.039	0.033
*Dt*_1–3_ (h)	18.06	19.43	16.81	20.13	22.04	18.45	19.21	20.73	17.85	19.33	20.80	18.00
R^2^	0.9958			0.9935			0.9953			0.9957		
S_yx_	0.1389			0.1764			0.1519			0.1435		

As observed in Table 1, CAP (air) appeared to be the most comparable to the control from other CAP treatments while also seemed to fair better from all plasma (CAP/PAW) treatments by demonstrating the highest growth rate of sprout stem (0.037 1/h) and smallest doubling time (18.73 h). Based on this assessment CAP (air) treatment of seeds was selected for the combined plasma treatment assessments.

#### Combined CAP and PAW treatment of seeds

A regression analysis of the averaged stem length of 20 mung bean sprouts for each of the three sample replicates recorded at various time intervals over 96 h of growth was performed (Supplementary Fig. S1). Throughout the 96 h experiment seeds were grown on a bed of glass beads, irrigated with 3 mL of PAW contained in test tubes and kept incubated in the dark at 25 °C ± 1 °C at 45% humidity. The growth rate and doubling time were obtained using the exponential growth equation and reported in Table 2.

**Table 2:** Growth rate constant (k) and doubling time (Dt) of mung bean sprouts of sample replicates 1–3 for each combined plasma treatment obtained from exponential growth equation.

	Control			CAP + PAW (air)			CAP + PAW (N_2_)			CAP + PAW (CO_2_)		
		CI_upper_	CI_lower_		CI_upper_	CI_lower_		CI_upper_	CI_lower_		CI_upper_	CI_lower_
*k*_1–3_ (1/h)	0.033	0.035	0.031	0.033	0.034	0.031	0.034	0.036	0.031	0.032	0.034	0.030
*Dt*_1–3_ (h)	20.77	22.09	19.57	21.28	22.50	20.16	20.67	22.12	19.35	21.70	23.24	20.31
R^2^	0.9709			0.9864			0.9800			0.9793		
S_yx_	0.2877			0.2140			0.2618			0.2287		

Goodness of fit represented by R squared (R^2^) value. Profile likelihood of each value demonstrated using 95% Confidence Interval (CI).

As observed in Table 2, the growth rate of mung bean sprouts appeared to be relatively similar with only a 0.001 1/h difference between the control and the combined plasma treatments in N_2_ [CAP + PAW (N_2_)] and in CO_2_ [CAP + PAW (CO_2_)]. In comparison to the control, the *Dt* varied by + 0.77 h, − 0.10 h and + 0.93 h for the combined plasma treatments in air [CAP + PAW (air)], N_2_ and CO_2_ respectively. However, statistical analysis determined no significant difference between the *k* and *Dt* values of the control to the treated groups as the p values (P) exceeded the 0.05 minimum level of significance in each case.

**Figure 1:**
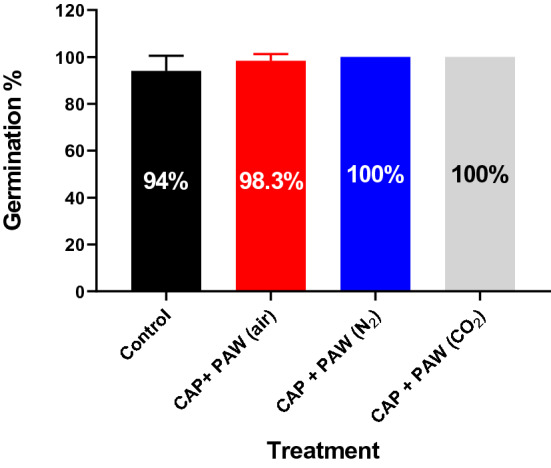
Percentage of mung bean seeds sprouted within 96 h under different combined plasma treatments.

Mung bean seed germination percentage, i.e., the percentage of seeds that sprouted by the end of the 96 h experiment under the different treatments are presented in Fig. 1. While slight increases in germination percentage were demonstrated by the combined plasma treatments in air (+ 4.3%), in N_2_ (+ 6%) and in CO_2_ (+ 6%) compared to the control, these increases were not found to be significantly different (P > 0.05).

**Figure 2:**
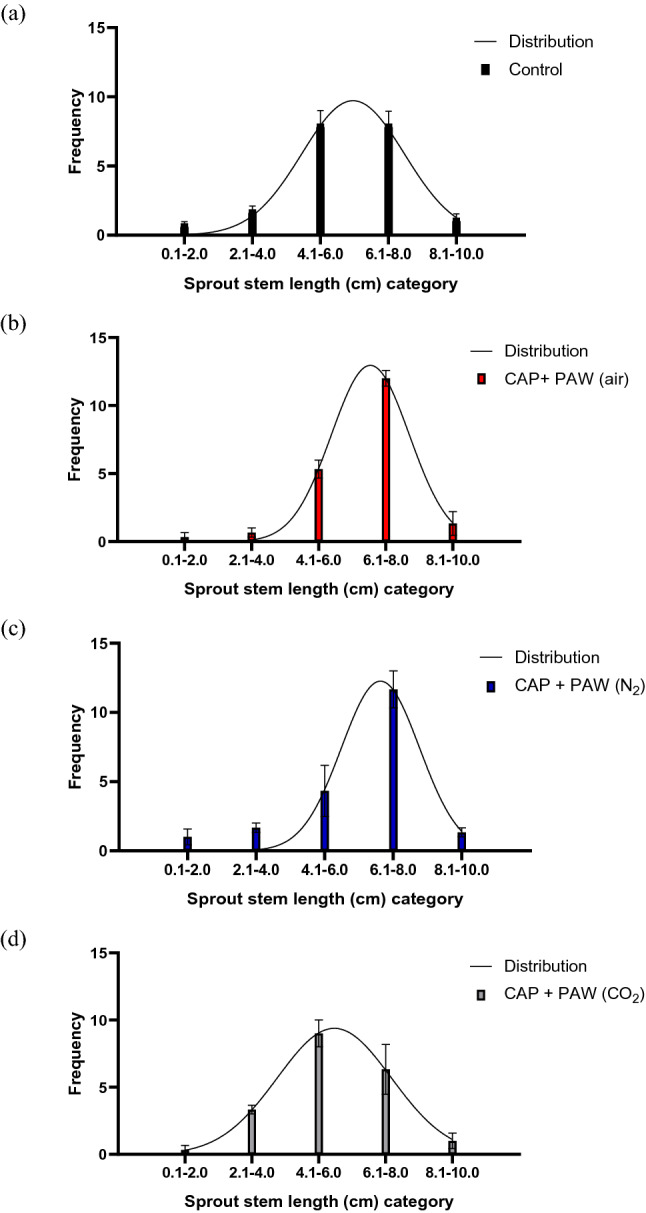
Frequency distribution of mung bean growth performance of the (**a**) control, (**b**) combined plasma treatment in air, (**c**) combined plasma treatment in N_2_ and (**d**) combined plasma treatment in CO_2_. Sprouts were categorized according to stem length achieved within 96 h of growth. A Gaussian curve was fitted to demonstrate distribution (R^2^ = (**a**) 0.7902, (**b**) 0.9607, (**c**) 0.8359, (**d**) 0.8418).

**Figure 3:**
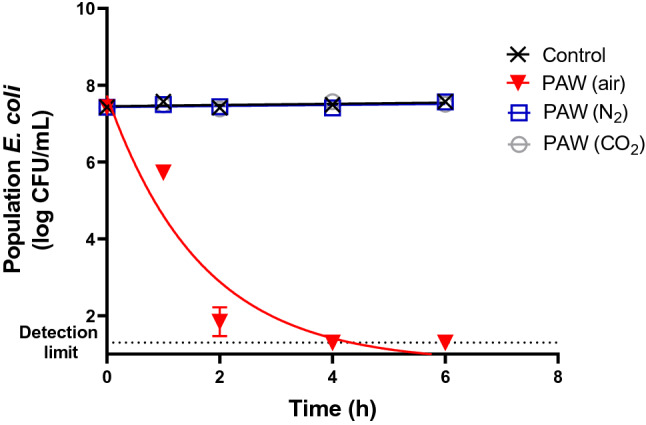
Inactivation of *E. coli* in relation to exposure time (h) to PAW (air), PAW (N_2_) and PAW (CO_2_). The exponential decay equation was used to analyse the dataset of the sample replicates of PAW (air) (R^2^ = 0.913).

Figure 2a–d demonstrate the distribution of sprouts according to the stage of growth, in terms of stem length (cm) achieved by the end point of the experiment, i.e., 96 h. Stage of growth is represented by five categories of stem length (cm) on the x-axis (0.1–2.0 cm, 2.1–4.0 cm, 4.1–6.0 cm, 6.1–8.0 cm and 8.1–10.0 cm). A Gaussian curve was fitted to give an overview of the performance of the sprouts in relation to the combined plasma treatment applied and to compare frequency distributions between treatments (Fig. 3). In contrast to the 39% of sprouts from the control group that achieved an end-point stem length between 6.1 and 8.0 cm, 60%, 58.5% and 31.5% of sprouts from the combined plasma treatment groups in air, N_2_ and CO_2_, respectively grew within the same category within 96 h. However, when using statistical analysis to compare the frequencies at the respective growth stages of the control to those of the combined plasma treatments, no significant difference (P > 0.05) was determined between either of the growth stages of each treatment. For the 6.1–8.0 cm stem length category, although the combined plasma treatments in air and N_2_ (as seen in Fig. 3) appeared to have a higher frequency than the control, no significant difference was observed with a P-value of 0.1040 and 0.1400, respectively.

### Overall characteristics of CAP and PAW processing

The averaged pH of the PAW samples produced throughout the study were 3.34 ± 0.07, 3.91 ± 0.05 and 3.64 ± 0.09 for PAW (air), PAW (N_2_) and PAW (CO_2_), respectively. The temperature of the PAWs was monitored after treatment and was never found to exceed room temperature.

### Characterisation of the irrigation plasma activated water

The determination of NO_2_^−^ and NO_3_^−^ concentrations provides an insight of the RONS species present within the PAW waters following irrigation and germination of mung bean seeds. The concentration of nitrate and nitrite species (mg/L) in the irrigation waters collected after 48 h and 96 h of germination are shown in Table 3. The irrigation waters of PAW (air) and PAW (CO_2_) demonstrated significantly greater concentrations of nitrates than the other irrigation waters of the control and of PAW (N_2_). In all samples, the level of nitrates in the irrigation waters reduced at 96 h that is the end of the germination assessment period. In the case of nitrites, the irrigation water of PAW (air) again shows to carry the highest level of nitrites followed by the irrigation of water of PAW (N_2_).

**Table 3:** Concentration (mg/L) of nitrates and nitrites in the irrigation PAWs collected after 48 h and 96 h of seed germination.

	Nitrate concentration (mg/L)		Nitrite concentration (mg/L)	
	48 h	96 h	48 h	96 h
Control	0.12 ± 0.02	0.04 ± 0.01	< 0.02	< 0.02
PAW (air)	2.72 ± 0.27	2.10 ± 0.30	2.69 ± 0.71	1.26 ± 0.80
PAW (N_2_)	0.44 ± 0.06	0.12 ± 0.05	0.82 ± 0.13	0.16 ± 0.11
PAW (CO_2_)	2.72 ± 0.54	1.70 ± 0.25	0.02 ± 0.02	0.01 ± 0.02

### Antimicrobial capacity of PAW

**Figure 4:**
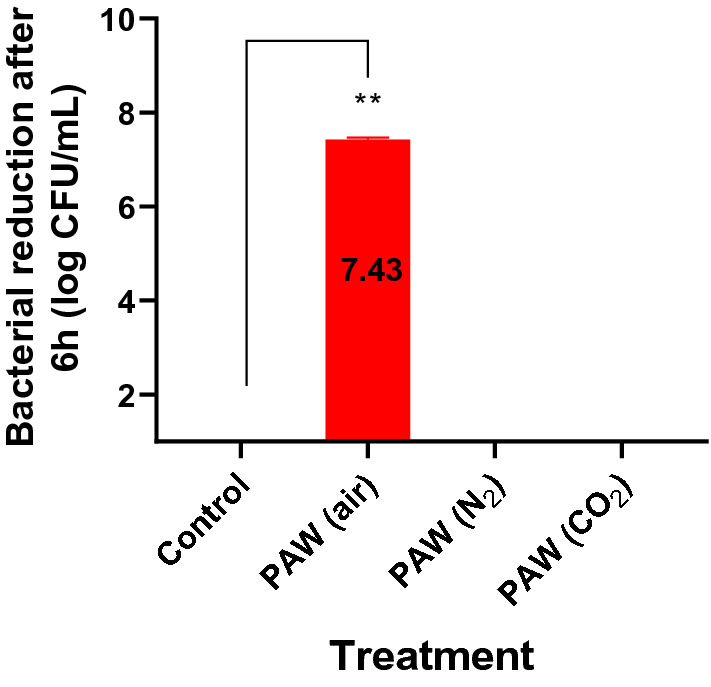
Total reduction of *E. coli* (log CFU/mL) within 6 h of exposure to different PAWs. **P ≤ 0.01.

The impact of PAW on *E. coli* (log CFU/mL) is depicted on Fig. 3. Evidently, a microbial reduction that went below the detection limit (1.3 log CFU/mL) was achieved within 4 h while the population of *E. coli* when exposed to the control, PAW (N_2_) and PAW (CO_2_) was steady and between 7 and 8 log CFU/mL. The kinetic data were analysed with an exponential decay equation from which the inactivation rate, expressed as rate constant (*k*) values (1/h) of *E. coli* exposed to PAW (air) and half-life (*Hl*) was determined (refer to Table 4). Figure 4 illustrates the total bacterial reduction of *E. coli* (log CFU/mL) within 6 h of exposure to each treatment. Significant reduction (P = 0.0013) in the population of *E. coli* exposed to PAW (air) (CI_upper_: − 7.046 log CFU/mL; CI_lower_: − 7.501 log CFU/mL) was found when compared to the control.

**Table 4:** Inactivation rate constant (k) and half-life (Hl) of *E. coli* exposed to PAW (air) replicates 1–3 obtained from the exponential decay equation.

	PAW (air)		
		CI_upper_	CI_lower_
*k*_1–3_ (1/h)	0.60	0.9262	0.3408
*Hl*_1–3_ (h)	1.16	2.034	0.7484
R^2^	0.913		
RMSE	0.8475		

Goodness of fit represented by R squared (R^2^) value. Profile likelihood of each value demonstrated using 95% Confidence Interval (CI).

#### Decontamination efficiency of CAP and PAW treated sprouts

**Figure 5:**
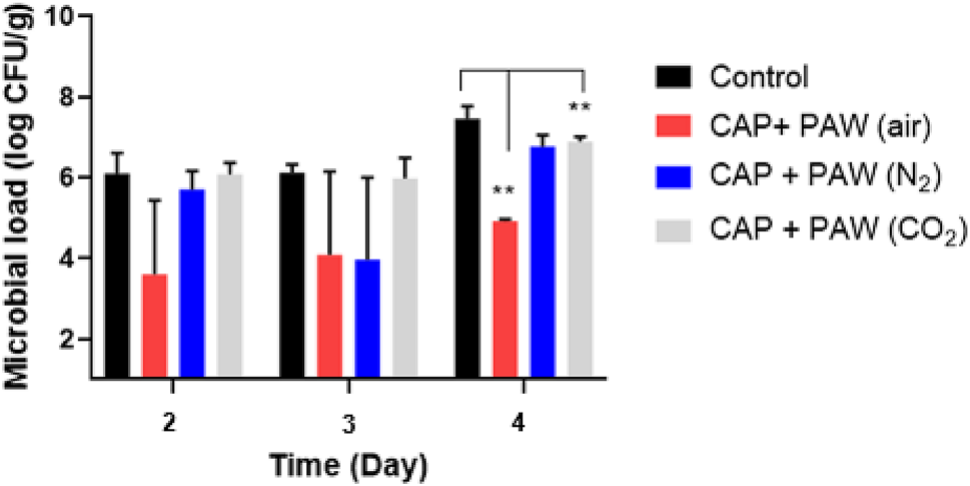
Total microbial population (log CFU/g) within sprouts of control and combined plasma treatments measured over 4 days of growth in plant chambers set at 25 °C ± 1 °C in the dark at 45% humidity level. **P ≤ 0.01.

The starting microbial population in the seeds was 2.79 ± 0.33 log CFU/g and 2.72 ± 0.60 log CFU/g for the control and CAP treated seeds, respectively. The microbial population (log CFU/g) of the combined plasma treated sprouts recorded on day 2, 3 and 4 of the sprout growth is depicted in Fig. 5. Sprouts of the CAP and PAW (air) treatment group maintained the best control over microbial proliferation throughout the 4 days of growth, measured at 4.92 log CFU/g on day 4, compared to the control sprouts (7.46 log CFU/g) and combined plasma treatments in N_2_ and CO_2_ (6.78 and 6.90 log CFU/g respectively). In fact, on day 4, the microbial population within the sprouts of the combined plasma treatments in air (P = 0.0053) and CO_2_ (P = 0.0092) were found to be significantly lower than that of the control. The variation between sample replicates may be attributed to biological variation between the sprouts and may have been reduced with further process replications.

### Analysis of secondary metabolites

**Figure 6:**
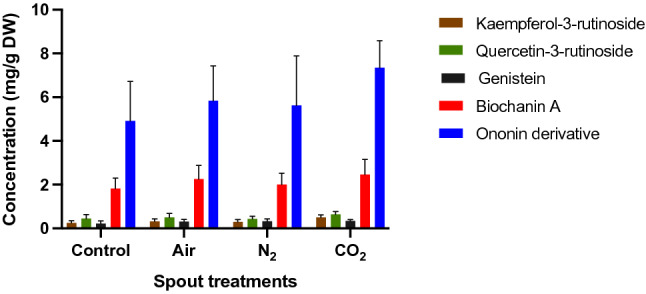
Impact of different plasma treatments on the secondary metabolites of sprouts expressed in dry weight (DW).

The concentration of different flavonoids in the sprouts is depicted in Fig. 6, where results are presented as mg/g dry weight. Overall, the concentration of secondary metabolites is relatively similar between sprouts from the control and the different combined plasma treatments with no significant disparities.

## Discussion

The interactions between the plasma generated RONS and water molecules have been found to enrich water with biochemically active species. These species demonstrate significant disinfection capacity^33,36,37^ and seed germination enhancing properties^28,38,39^, which appear to be more pronounced than when generated during direct CAP treatment over a longer exposure time. The latter treatment plays a significant role in the surface modification of the seed coat, by increasing its surface wettability and increasing the absorption of water into the seed^29^. The acidic environment generated in PAW (air) (pH 3.34 ± 0.07) correlates with the higher levels of NO_3_^−^ species quantified in PAW (air), as HNO_3_ is one of the leading agents causing acidification of PAW. Relatively higher pH values were observed in PAW (N_2_) (3.91 ± 0.05) and PAW (CO_2_) (3.64 ± 0.09) as the quantity of reactive nitrogen species decreased.

Similar to the germination assessment outcomes of individual CAP and PAW treated mung bean seeds, the three combined CAP and PAW treatments showed no significant stimulatory effect on mung bean seed germination or performance, although as relevant is the lack of inhibitory effect. This outcome contradicts the body of research evidencing significant increases in germination rate following cold atmospheric pressure plasma treatment of seeds^40–44^. For instance, Zhou et al.^45^ found that air plasma produced through DBD microplasma array (4.5 kV; 25 W) offered the best efficiency in improving mung bean seed germination rate and seedling growth when compared to the control and O_2_, N_2_ and He microplasma arrays. However, contrary to the germination conditions used in this study (incubation of seeds in test-tubes at 25 °C ± 1 °C in the dark at 45% humidity level with water replacement after 48 h), Zhou et al.^45^ kept the seeds in Petri dishes, watered them daily and incubated them in light conditions. In another study, CAP produced using a DCSBD at 400 W with a plasma volume power density of 70 W/cm^3^ was employed to treat wheat seeds^46^. While these conditions are similar to those applied in this study, the wheat seeds were placed directly on the ceramic plate of the DCSBD device and fixed to a rotation device to homogeneously treat the seed surfaces^46^. Contrary to this, mung bean seeds assessed in this study were treated bi-directionally in a static position between two plasma plates kept at a distance of 1.5 cm each from the seeds. Furthermore, germination and growth of wheat seeds was assessed by sowing the seeds in pots containing soil substrate (a mixture of sand, peat and pearlite). The wheat seeds exhibited a greater uptake of water than their untreated counterparts. In fact, following 20–50 s CAP-air treatment of seeds, significant accession of germination rate, dry weight and vigour of wheat seedlings was observed^46^. In comparing studies, germination conditions using light and soil are not realistically associated with sprout production practices. However, while taking into account that the closed germination set-up used in this study (test tubes) facilitated the microbiological assessments performed to determine the decontamination efficiency of combined plasma treated sprouts, the same system is not realistically comparable to common sprout production practices that enable the regular irrigation of sprouts. Furthermore, in comparing the technical plasma treatment parameters between studies, the distance of the seeds to the plasma source and associated treatment time during CAP treatment of seeds along with the frequency of irrigation with PAW may be key factors in regulating the level of exposure of seeds to reactive species that in turn determines the efficacy of plasma treatment on seed germination and growth.

Few studies have looked into the combined effect of direct CAP treatment of seeds and irrigation with PAW on seed germination and growth. In one study, Sivachandiran and Khacef^28^ elucidated the short term effects of combined plasma treatment on seed germination and stem growth of radish sprouts, and the long term effects of combined plasma treatment on tomato and sweet pepper plant growth. The study made use of a plate-to-plate DBD (operated with a high voltage pulsed power of 40 kV and frequency of 1 kHz) for the CAP treatment of seeds in air for 10 min (P 10) and 20 min (P 20) and a cylindrical DBD (40 kV; 1 kHz) for the cold atmospheric plasma activation of water in air for 15 (PAW 15) and 30 min (PAW 30). Seeds of the P 10 treatment irrigated with PAW 15 displayed better seedling growth when compared to the untreated seeds and to the P 10 treated seeds irrigated with tap water. Furthermore, non-treated seeds irrigated with PAW 30 performed better than non-treated seeds irrigated with tap water. Through this outcome, it was observed that aside from the stimulatory effect of plasma discharge on seed surface, sustained plasma treatment through irrigation of treated seeds with PAW can significantly enhance seedling growth. As may be applied in this study, achieving enhanced seed germination and seedling growth would require optimization of combined plasma treatment for each seed type, taking into account both gas phase characterisation and the physicochemical properties of the PAW^28^.

When assessing the inactivation of *E. coli* following exposure to PAW (air), PAW (N_2_) and PAW (CO_2_), it was found that although all three PAWs exhibited an acidic pH (3.34–3.91), only PAW (air) was found to inactivate *E. coli*. This suggests that an acidic environment is unlikely to be the sole sterilizing agent associated with PAW treatment, but that the type and concentration of reactive species within the PAWs also play a significant role in determining its biological effect. In fact, peroxynitrite, a nitrogen containing reactive species, was previously found to play a crucial role in the antibacterial application of PAW (air) due to its cytotoxic effects^36,38^. Peroxynitrite is mostly formed by the reaction between H_2_O_2_ and NO_2_^38^. Theoretically, this reaction would not be possible in PAW (CO_2_) for lack of nitrogen in the process gases, nor in PAW (N_2_) for lack of reactive oxygen species such as H_2_O_2_ during plasma discharge, while it can be the case for the PAW (air). Further insight into the effect of the reactive species could be gained through independent inactivation assessments of *E. coli* following exposure to simulated aqueous concentrations of the reactive species identified and quantified in the PAW (e.g. H_2_O_2_, NO_3_^−^, NO_2_^−^, ONOO^−^, O_3_, and OH^−^). Another study aimed to elucidate the bacterial cell damage caused to *Staphylococcus aureus* by oxidative stress from PAW generated in a single electrode alternating current cold plasma set-up^47^. Among the techniques used, atomic absorption spectroscopy detected an increased leakage of potassium ions from bacterial cytoplasm and transmission electron microscopy revealed morphological impairments to bacterial cell wall and membrane following exposure to PAW. The same paper also indicated the effect of short-lived species within PAW, noting that a bacterial suspension of *S. aureus* exposed to PAW directly after production and the same PAW stored in a 4 °C refrigerator for 24 h after production required 10 min and 40 min, respectively, to achieve a 6 log reduction in the population of *S. aureus*^47^. The aforementioned techniques may also be employed to better substantiate and characterise the antimicrobial properties of PAW.

The lifetime of reactive species is an important parameter in understanding the long-term decontamination effects of PAW treatment on seeds. An initial screening on sprouts following CAP and PAW treatment of mung bean seeds indicated that CAP (air) treated seeds irrigated with PAW (air) demonstrated the greatest control over the natural microbiota (4.92 log CFU/g) of the sprouts after 96 h in the incubation chambers (25 °C) when compared to the control and other combined plasma treated groups (> 6 log CFU/g). This outcome potentially enhances the relevance of combined plasma treatment as a means of inhibiting bacterial proliferation during sprout production, which is stimulated by the warm temperatures, water activity and high nutritive content in the sprouts. The increased decontamination efficiency of the combined plasma treatment in air may correspond to the antimicrobial effect of PAW (air) determined in respect to *E. coli*, however further characterisation, primarily through identification of microorganisms is required. To the best of our knowledge, no previous studies have monitored the microbial population within sprouts following CAP and PAW treatment of seeds. However, recent studies have assessed the use of PAW as a means of decontaminating harvested mung bean sprouts with ancillary evaluation of the physicochemical characteristics of the sprouts following treatment. Schnabel et al.^48^ assessed the inactivation rates of *E. coli*, *Pseudomonas fluorescens*, *Pseudomonas marginalis* and *Pectobacterium carotovorum* in sprouts after 5 min immersion in air plasma processed water. The highest inactivation rates were observed for *E. coli* and *P. marginalis* while minimal effect on texture and appearance of the sprouts was recorded. More recently, Xiang et al.^29^ found that the total aerobic bacteria and yeasts within mung bean sprouts decreased by 2.32 and 2.84 log CFU/g, respectively following 30 min immersion of sprouts in air PAW. The authors also established that the washing treatment had no significant effect on antioxidant potential of mung bean sprouts and no changes in the phenolic and flavonoid contents nor sensory characteristics of the sprouts.

In the mung bean sprouts, kaempferol-3-rutinoside, quercetin-3-rutinoside, genistein, biochanin A and ononin derivative were tentatively identified. A review on the phytochemical profile of mung bean sprouts mentions other phenolic compounds and flavonoids present in the sprouts such as catechin, syringic acid, gallic acid, vitexin, robinin, kaempferol-7-O-rhamnoside and isoquercitrin^49^ which were not found here. A variation of flavonoids and phenolic acids is common in *Vigna* species. in a previous study on pea sprouts exposed to CAP resulted in highest concentrations of quercetin and kaempferol glycosides, whereas the treatment of seeds and seedlings was not as efficient or even decreased quercetin and kaempferol glycosides^50^. Here all combined plasma treatments had no significant impact on the concentrations of secondary metabolites in the sprouts that gives a positive outcome on the application of cold plasma treatment in the agro-food industry. Future work could focus on analysing the phytochemical profiles of the sprouts pre and post-treatments.

Overall, this research demonstrated that air plasma activated water formed by 1 min surface treatment with cold atmospheric plasma generated at 350 W (at a frequency of 15 kHz) in the DCSBD was able to reduce the population of *E. coli* DSM1116 by 7.43 log CFU/mL within 6 h of exposure. The combined seed treatment with direct air CAP (5 min, 350 W, with a power density up to 100 W/cm^3^) and air PAW had no negative impact on mung bean seed germination and growth when compared to the control, nor was the concentration of secondary metabolites within the sprouts reduced. The combined air CAP and air PAW treatment reduced the total microbial population in sprouts by 2.5 log CFU/g lower than the population of the control within 4 days, although further characterisation of the natural microbiota on the seeds prior to and after cold plasma treatment would allow for more accurate assessments on the effect of prolonged plasma treatment on the sprouts. The concentration of long-lived reactive nitrogen species, such as NO_3_^−^, NO_2_^−^ are crucial in elucidating the complex mechanisms of action of PAW in disinfection and germination, along with the further characterisation of other reactive species such as hydrogen peroxide. Finally, microscopic imaging could also allow to assess any surface modifications that occur on the processed seed coats while measuring the absorption time of a water droplet would contribute to surface wettability evaluations.

## Materials and method

### Plasma source

The operating conditions for the Diffuse Coplanar Surface Barrier Discharge (DCSBD) plasma system (CEPLANT, Brno, Czech Republic) used in this study were described in a previous study^51^ and are summarized below. This plasma system allowed for the operation of a homogeneous plasma produce in the presence of a process gas. For the purpose of this research three process gases were employed: air; N_2_; and a mixture of 80% CO_2_ with 20% O_2_. The DCSBD system consisted of two parallel opposing plates, the distance between which can be adjusted in the range of 0–30 mm. Strip-like electrodes (1.5 mm width, 0.5 mm thickness and 1 mm distance between electrodes) generate plasma on the surface of each plate. The electrodes do not come into contact with the plasma as they are separated by a 0.4 mm thick ceramic layer made of 96% alumina. A dielectric insulating oil circulation system was used for the electric insolation of the electrodes. The electrode arrangement of the DCSBD is presented in Hertwirg et al.^52^.

An homogenous plasma layer is generated over an area of 200 mm × 80 mm on the electrode surface with an increase on the power. The plasma has a thickness of 0.3 mm and operates through high frequency (15 kHz) sinusoids, with a voltage up to 20 kV (peak to peak) and power density up to 100 W/cm^3^. An external cooling system (Huber CC-410, Offenburg, Germany) is connected and set to 15 °C to ensure the insulating oil temperature did not exceed 65 °C.

### Seed treatments and assessment of germination and growth

The study complied with local and national regulation.

#### Germination set-up

A test tube set-up, adapted from Darmanin et al.^8^, was used for germination assessments. Test tubes filled with 9 g of glass beads were sterilized at 121 °C for 15 min prior to use. One mung bean seed, *Phaseolus Mungo*, (procured commercially from Bavicchi, Perugia, (Italy) complying with relevant institutional, national, and international guidelines and legislation) was inserted per tube and then irrigated with 3 mL (enough to partially immerse the seed) PAW or sterile distilled water (SDW) as the negative control. The tubes were placed in incubation chambers set at 25 °C ± 1 °C in the dark at 45% humidity level for 96 h. Following the first irrigation with SDW/PAW (0 h), the water in the tubes was replaced with fresh SDW/PAW at 48 h in order to re-enrich the irrigation system. The length of the sprout stem was measured 3–4 times a day using ImageJ software 1.53b (https://imagej.nih.gov/ij/download.html), from 48 h (i.e., time that sprouting was initiated) until 96 h to calculate growth rate and compare growth performance. Germination percentage at end point was also recorded. The aforementioned seed germination and growth assessment technique was applied for all plasma treatments described below.

#### CAP treatment of seeds

**Figure 7:**
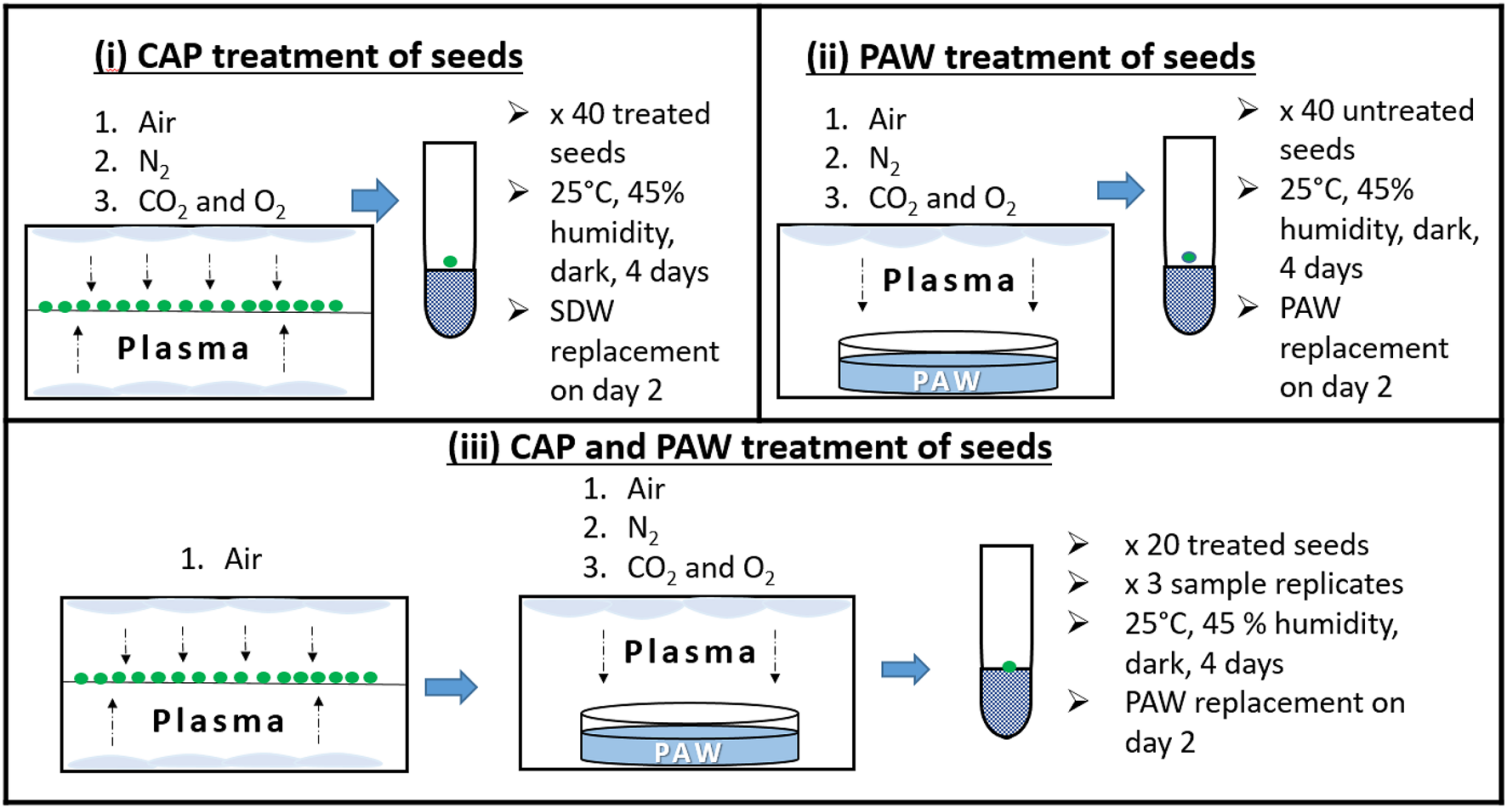
Summary of plasma parameters and experimental method adopted for assessing mung bean seed germination and growth following (**i**) CAP treatment of seeds, (**ii**) PAW treatment of seeds and (**iii**) combined CAP and PAW treatment of seeds.

The first set of experiments was performed to assess mung bean seed germination and growth following seed treatment with CAP (refer to Fig. 7i). The previously described DCSBD plasma device was used, where three CAP treatments were assessed that varied by process gas: CAP (air) treatment infused the DCSBD chamber with 10 SLM air for 2 min, CAP (N_2_) treatment infused the DCSBD chamber with 10 SLM N_2_ for 2 min and CAP (CO_2_) using a mixture of 80% CO_2_ (2 SLM) and 20% O_2_ (0.5 SLM) infused the DCSBD chamber for 5 min. For each treatment one sample set of 40 seeds (technical replicates) was treated bi-directionally in the DCSBD plasma chamber set at 350 W for 5 min at a distance of 1.5 cm both from the top and bottom plate. Single seeds were placed in 40 tubes, irrigated with SDW and incubated together with an untreated set which served as negative control.

#### PAW treatment of seeds

The second set of treatments assessed germination and growth of untreated seeds irrigated with 3 mL PAW (Fig. 7ii). The same process gas parameters as described under CAP treatment of seeds were applied for the generation of three different PAWs: PAW (air), PAW (N_2_) and PAW (CO_2_). The DCSBD was set to 350 W for the surface CAP treatment of 50 mL SDW for 1 min with a distance of 2 cm from the surface of the water to the top electrode. Following the treatments, pH and temperature of the PAWs was recorded. Hereafter, 40 tubes with single untreated mung bean seeds were irrigated with 3 mL of PAW and were incubated together with an untreated set that served as negative control.

#### Combined CAP and PAW treatment of seeds

Based upon assessment and evaluation of the individual effect of CAP and PAW treatments on mung bean growth rate and growth performance, one CAP treatment of seeds was combined with each of the three types for PAWs (Fig. 7iii). The three combined plasma treatments assessed were: CAP (air) treated seeds irrigated with PAW (air), CAP (air) treated seeds irrigated with PAW (N_2_) and CAP (air) treated seeds irrigated with PAW (CO_2_). As all CAP treatments were performed in air, the annotations for the combined plasma treatments took on the following format: CAP + PAW (air), CAP + PAW (N_2_) and CAP + PAW (CO_2_). Each combined plasma treatment was assessed in triplicate on a set of 20 mung bean seeds. A triplicate set for the negative control was also performed simultaneously.

### Characterisation of the irrigation plasma activated water

Following irrigation of seeds with PAW, the irrigation water was collected after 48 h and 96 h of germination. The water samples were pooled and the concentration of nitrite (NO^2−^) and nitrate (NO^3−^) species in PAW samples were determined using an ICS-1000 ion chromatography system (Thermo Scientific Dionex, Germany), with a sample injection volume of 25 µL. The anions were eluted using a 4 × 250 mm IonPac AS9-HC anion-exchange column (Thermo Fisher DIONEX, Germany), equipped with an ASRS-Ultra detector. The eluent was Na_2_CO_3_ (9 mmol) and the flow rate was 1.2 mL/min.

### Determination of the antimicrobial capacity of PAW

The antimicrobial capacity of the PAWs was determined by assessing the inactivation of *Escherichia coli* DSM1116 over 6 h exposure to PAW. A two-step re-culturing of *E. coli* from a bead culture stored at − 80 °C was performed by transferring 1 bead to 5 mL Nutrient Broth (NB; ROTH, Karlsruhe, Germany). This suspension, termed as the pre-culture was vortexed and incubated at 37 °C for approximately 24 h. The second sub-culture was incubated at 37 °C for approximately 18 h until stationary phase was reached, whilst shaking at 165 rpm. A Multisizer (Beckman Coulter, California, US) particle counter was used to obtain a starting concentration of approximately 10^8^ CFU/mL. Hereafter, 1 mL of the prepared culture was exposed to 9 mL of PAW while 9 mL sterile distilled water was used as a negative control. For each PAW sample, two technical replicate samples were analyzed after 0 (control only), 1, 2, 4 and 6 h of PAW exposure. Analysis was performed by diluting the sample in Ringers solution inside a microtiter plate. Selected dilutions were plated on Plate Count Agar (PCA, ROTH, Karlsruhe, Germany). Plates were incubated at 37 °C and bacterial counts were recorded after 24 h to calculate inactivation rate and compare log bacterial reductions between treatments. Apart from assessing two technical replicates from each sample, the procedure also assessed the antimicrobial capacity of three independent replicate samples of the control, PAW (air), PAW (N_2_) and PAW (CO_2_).

### Determination of the microbial load of CAP and PAW treated sprouts

Microbial assessments were performed on sprouts grown from the combined plasma treatment of seeds (CAP and PAW) in order to determine the effect of plasma treatment on the natural microbiota of the sprouts. CAP treated seeds were assessed in order to determine the starting microbial population within both specimens. Untreated seeds were assessed as negative control. Germination was initiated following irrigation of seeds with respective treatment (SDW/PAW) in the test tube set-up and incubated at 25 °C ± 1 °C in the dark at 45% humidity level for 4 days. The microbial population in the sprouts was assessed on day 2, 3 and 4.

Approximately 5 g of seed and sprout samples were first homogenized in a 1/10 dilution in buffered peptone water (ROTH, Karlsruhe, Germany). From each homogenate, two technical replicate samples were analyzed. Analysis was performed by diluting the homogenate in casein peptone water (ROTH, Karlsruhe, Germany) inside a microtiter plate. Selected dilutions were plated on PCA and incubated at 30 °C. Bacterial counts were recorded after 72 h in order to compare microbial load of the treated seeds and sprouts to the control. Apart from assessing two technical replicates from each homogenate, the procedure also assessed the decontamination efficiency of three independent replicate sample sets of control (seeds) and CAP treated seeds and control (sprouts) and combined plasma treated sprouts.

### Quantification of secondary metabolites

The method of extraction and quantification of secondary plant metabolites in the sprouts grown after 96 h of germination was adapted from Neugart et al.^53^. This involved dissolving 10 mg of freeze-dried sprouts in 600 µL 60% methanol and shaking for 40 min at 1400 rpm and 20 °C in a thermal shaker. Centrifugation at 4500 rpm and 20 °C for 10 min followed. The resulting supernatant was collected in a new reaction vessel. The pellet was dissolved again in 300 µL 60% MeOH and shaken at 1400 rpm and 20 °C for 15 min. The sample was centrifuged at 4500 rpm and 20 °C for 10 min. The supernatant was collected again. This step was performed twice. The collected supernatant was left to evaporate in the vacuum centrifuge until completely dry. The residue was dissolved in 200 µL 10% MeOH. The solution was then poured into Spin-X/Filter tubes with a 0.22 μm cellulose acetate membrane (Corning Costar Spin-X, Sigma Aldrich Chemical Co., St. Louis, MI). These were then centrifuged at 3000 rpm and 20 °C for 5 min. The remaining solution was then prepared for measurement with the high performance liquid chromatography mass spectrometry (HPLC–MS).

Flavonoid profile (including glycosides of flavonoids) and concentrations were determined from the filtrate using a series 1100 HPLC (Agilent Technologies, Waldbronn, Germany) equipped with a degaser, binary pump, autosampler, column oven, and photodiode array detector. A F5 column (Ascentis Express, 150 mm × 4.6 mm, 5 µm, Supelco) was used to separate the compounds at a temperature of 25 °C. Eluent A was 0.5% acetic acid, and eluent B was 100% acetonitrile. The gradient used for eluent B was 5–12% (0–3 min), 12–25% (3–46 min), 25–90% (46–49.5 min), 90% isocratic (49.5–52 min), 90–5% (52–52.7 min), and 5% isocratic (52.7–59 min). The determination was conducted at a flow rate of 0.85 mL/min and a wavelengths of 280 nm, 320 nm, 330 nm, 370 nm and 520 nm. The glycosides of flavonoids were identified as deprotonated molecular ions and characteristic mass fragment ions according to^50^ and^53^ by HPLC-DAD-ESI-MS^n^ using an Bruker amazon SL ion trap mass spectrometer in negative ionisation mode. Nitrogen was used as the dry gas (10 L/min, 325 °C) and the nebulizer gas (40 psi) with a capillary voltage of − 3500 V. Helium was used as the collision gas in the ion trap. The mass optimization for the ion optics of the mass spectrometer for quercetin was performed at m/z 301 or arbitrarily at m/z 1000. The MSn experiments were performed in auto up to MS3 in a scan from m/z 200–2000. Standards (quercertin 3-glucoside and kaempferol 3-glucoside, Roth, Karlsruhe, Germany) were used for external calibration curves in a semi-quantitative approach.

### Statistical analysis

Statistical analysis was performed using GraphPad Prism 8.0.1. The sprout stem length measurements recorded over time during the germination experiments were analyzed using the exponential growth equation. The exponential decay equation was used to analyze the log bacterial counts recorded over time during the PAW antimicrobial capacity experiments. Data sets including sprout stem growth rate, growth performance and germination percentage, log reduction of *E. coli* and log microbial population on sprouts were then tested for normality using the D’Agostino and Pearson test and the Shapiro Wilk test, performing data transformation when necessary. Statistical analysis using one-way ANOVA, two-way ANOVA and the Friedman test were performed to determine significance of treatment results compared to control results.

## Supplementary Information

Supplementary Figure S1.
