# Supplementary material for: Aqueous and gaseous plasma applications for the treatment of mung bean seeds
Source: Sci Rep. 2021 Oct 4;11:19681. doi: 10.1038/s41598-021-97823-1 (PMC8490402; doi:10.1038/s41598-021-97823-1)
Supplement: Supplementary file 1 — Supplementary Figure S1. [file 41598_2021_97823_MOESM1_ESM.docx]

| (a) |  |
| --- | --- |
| (b) |  |
| (c) |  |
| (d) |  |

**Supplementary Fig. S1.** Averaged length (cm) of 20 mung bean sprout stems over time (h), grown from (a) untreated seeds irrigated with SDW (control), (b) CAP (air) treated seeds irrigated with PAW (air), (c) CAP (air) treated seeds irrigated with PAW (N_2_) and (d) CAP (air) treated seeds irrigated with PAW (CO_2_). The exponential growth equation was used to analyse the datasets of the sample replicates.
